# A Novel Sequential Regimen of Efgartigimod and Telitacicept as Fast‐Acting Treatment for Impeding Crisis of MuSK‐MG

**DOI:** 10.1002/iid3.70521

**Published:** 2026-10-09

**Authors:** Tianjiao Duan, Song Ouyang, Wei Lu, Weifan Yin

**Affiliations:** ^1^ Department of Neurology, the Second Xiangya Hospital Central South University Changsha Hunan China; ^2^ Clinical Medical Research Center for Stroke Prevention and Treatment of Hunan Province, Department of Neurology, the Second Xiangya Hospital Central South University Changsha Hunan China; ^3^ Department of Neurology, the Affiliated Changsha Hospital of Xiangya School of Medicine Central South University Changsha Hunan China

**Keywords:** efgartigimod, impeding crisis, muscle‐specific kinase, myasthenia gravis, telitacicept

## Abstract

**Background:**

Myasthenia gravis (MG) is an autoimmune disorder of the neuromuscular junction. The muscle‐specific kinase (MuSK) antibody‐positive subtype often involves bulbar and respiratory muscles, conferring a high risk of myasthenic crisis.

**Method:**

We report a very late‐onset MuSK‐MG case complicated by lung infection and acute heart failure, with a history of peptic ulcer. The patient was sequentially treated with efgartigimod and telitacicept, achieving minimal symptom expression (MSE) within 1.5 months.

**Discussion:**

Following the first efgartigimod infusion, QMG and MG‐ADL scores improved from 31 to 23 and 22 to 12, respectively. After five injections of telitacicept, symptoms resolved completely on day 47, with scores further reducing to 2 (QMG) and 0 (MG‐ADL). Treatment also reduced IgG levels and MuSK‐Ab titers, while IgM remained stable.

**Conclusion:**

This case demonstrates the successful sequential use of efgartigimod and telitacicept in managing complicated, late‐onset MuSK‐MG in a state of impeding crisis.

## 1 Introduction

Myasthenia gravis (MG) is an autoimmune disorder affecting the neuromuscular junction, leading to fluctuating muscle weakness. Most cases involve autoantibodies against the acetylcholine receptor (AChR), while a minority target muscle‐specific kinase (MuSK) or lipoprotein‐related protein 4 (LRP4) [1]. MuSK‐MG, presenting in 5%–8% of MG patients, predominantly affects bulbar and respiratory muscles, frequently resulting in myasthenic crises (MC) and respiratory failure [1, 2, 3]. The impeding myasthenic crisis is the early stage of myasthenic crises, developing into a crisis within days or weeks [2]. The clinical judgment criteria for this stage are based on rapidly progressing symptoms of medulla oblongata or respiratory muscle. Accurate assessment is quantified by quantitative MG (QMG) score: ≥ 3 points of medulla oblongata subitem; or ≥ 2 points of respiratory muscle subitem; or ≥ 4 points of medulla oblongata plus respiratory muscles [4]. Timely identification and treatment of this condition is expected to block the disease process, therefore, reducing the occurrence of MC.

Traditional therapeutic regimens, like pyridostigmine, thymectomy, and intravenous immunoglobulin (IVIG) exhibit limited efficacy in MuSK‐MG patients [5], while plasma exchange (PLEX) and immunoadsorption can achieve symptom control rapidly in acute settings, but their utility is constrained by the need for invasive vascular access. Immunosuppressants, which act slowly, are often combined with corticosteroids; however, prolonged corticosteroid use increases the risk of serious side effects, particularly in elderly patients [5]. Similarly, rituximab, by depleting CD20‐positive B cells, confers a substantial infection risk [6]. Given that the median time to reach minimal symptom expression (MSE) in MuSK‐MG patients ranged from 11 weeks to 10.5 months with immunosuppressants or rituximab [7], a newer approach is needed to shorten the time to achieve MSE while ensuring safety.

FcRn inhibitors, which prevent FcRn from interacting with IgG, resulted in IgG clearance and are used as treatment for IgG‐mediated diseases [8]. Efgartigimod is the first FcRn inhibitor approved for AChR‐MG, demonstrating a fast therapeutic effect of clinical improvements in the ADAPT study within 2 weeks, accompanied by a rapid and large decrease in all IgG subtypes [9]. Telitacicept, a novel recombinant fusion protein, was designed to inhibit the activity of two target cytokines, the B cell lymphocyte stimulator (BlyS) and a proliferation‐inducing ligand (APRIL). Both the two targets are associated with B cell maturation, activation, and differentiation [10]. In its phase 3 clinical trial for MG treatment, the MG‐specific activities of daily living scale (MG‐ADL) score of therapeutic patients decreased by 5.74 points, the QMG score decreased by 8.66 points at week 24, and the indication has been approved for AChR‐positive generalized MG in China. Nonetheless, the evidence for efficacy and safety in MuSK‐MG patients remains limited. To date, clinical reports or articles on the treatment of MuSK‐MG with sequential efgartigimod and telitacicept are lacking. Since the mechanisms of these two drugs are different, the effect of the combination is worthing expectation.

In this case, we report a very late‐onset MuSK‐MG patient, who has complications of severe lung infection and acute heart failure. This patient was sequentially treated with efgartigimod and telitacicept, and achieved MSE within 1.5 months. Elderly patients with complex comorbidities in MuSK‐MG are still scarcely reported. Meanwhile, an avenue to rapidly reach MSE and stable remission in very late‐onset MuSK‐MG patients remains a therapeutic challenge.

### 1.1 Case Report

A 73‐year‐old male patient was admitted in July 2025 due to a 1‐month history of diplopia and dysphagia, accompanied by 1 week of fatigue and shortness of breath. A gastric tube was inserted after admission. Blood gas analysis showed no obvious dioxide retention temporarily. Previous history includes gastrointestinal ulcers, hypertension, diabetes, and coronary heart disease. Magnetic resonance imaging (MRI) was performed, and brain lesions were excluded. Although electromyography showed no reduction with low‐frequency electrical stimulation, subsequent serology antibody test was positive for MuSK‐Ab (1:100, cell‐based assay) and negative for AChR‐Ab. The initial evaluation by QMG score was 31 points, and MG‐ADL score was 22 points. The scores of bulbar plus respiratory muscles reached 9 points by QMG; this patient was diagnosed with MuSK‐MG in a state of impeding myasthenia crisis. Moreover, the patient had an obvious cough and expectoration on admission. Chest computed tomography (CT) revealed an infectious inflammatory lesion in the middle lobe of the right lung and bilateral pleural effusion (Figure 2A). Levels of leukocytes, C‐reactive protein, erythrocyte sedimentation rate, and procalcitonin in blood were high, and then sputum culture showed Klebsiella pneumoniae infection. Bone density suggests osteoporosis. Two days after admission, he developed worsening shortness of breath and could not lie flat at night. The cardiovascular specialist considered cardiomegaly and acute exacerbation of chronic heart failure.

After evaluating the age factor, infection risk, and underlying cardiac disease of the patient, we considered high‐dose glucocorticoids, IVIG, and PLEX to be inappropriate. In the acute setting, efgartigimod was administered to alleviate symptoms. Following two injections of efgartigimod (400 mg/week), subcutaneous therapy with telitacicept (240 mg/week) was initiated after a 1‐week interval. Meanwhile, antibiotics combined with natriuretic peptides were used to control infection and heart failure. The flowchart of the treatment regimen was shown in Figure 1. Five days after the first use of efgartigimod, rapid improvement of limb weakness was observed with QMG score of 23 and MG‐ADL score of 12. After the second dose of efgartigimod, the patient had the gastric tube removed and resumed independent feeding. The change in each component of the QMG scores at different time points was shown in Table 1. Before the first administration of telitacicept, lung infection improved significantly (Figure 2B). Following two injections of telitacicept on day 28, the patient's dysphagia and weakness resolved completely, with only slight diplopia in the horizontal gaze. He was discharged with a QMG score of 10 and an MG‐ADL score of 8. Telitacicept weekly subcutaneous injection was sustained, and the patient came back to outpatient for follow‐up after five injections on day 47. Daily life has completely returned to normal with a QMG score of 2 (low lung capacity) and an MG‐ADL score of 0 (Figure 3A). The telitacicept regimen was maintained with an extended injection interval of every 10 days. The levels of IgG and IgM were monitored before and after therapy, with a marked reduction in IgG and almost unaffected IgM (Figure 3B). The MuSK‐Ab titers also decreased after 1 month of treatment with telitacicept (Figure 3C).

**Figure 1:**
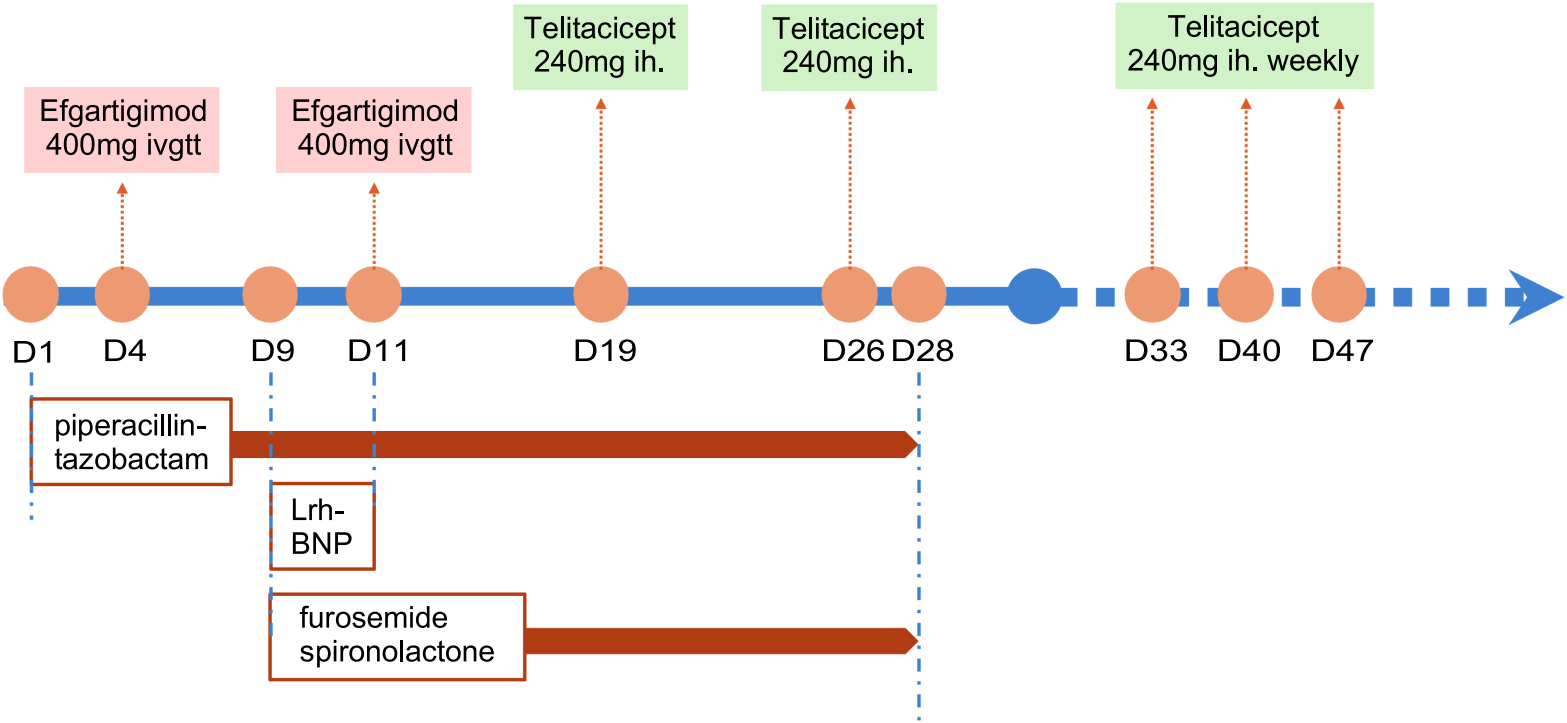
The flowchart of immunotherapy combined with anti‐infection and anti–heart failure treatment in the MuSK‐MG patient.

**Figure 2:**
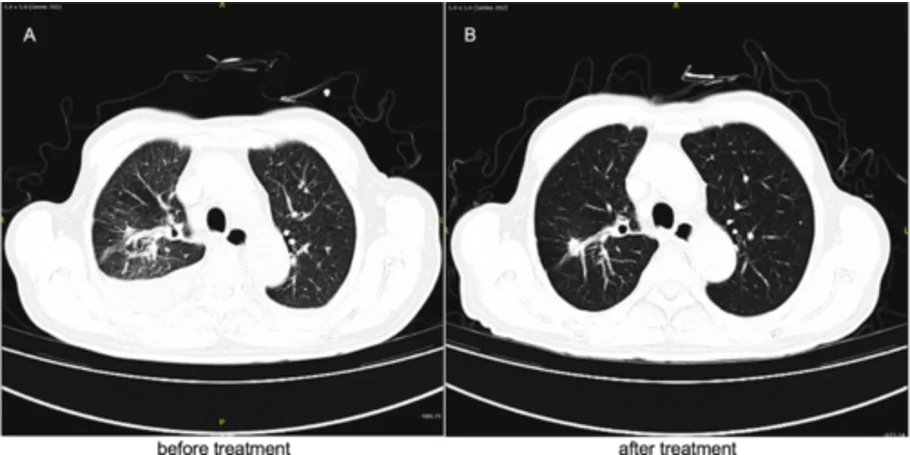
The comparison of lung CT images before (A) and after (B) treatment.

**Table 1:** The change in each component of the QMG scores at different time points.

Test item	D3	D9	D28	D47
Double vision	3	3	2	0
Ptosis	0	0	0	0
Facial muscles	3	2	2	0
Swallowing	3	3	1	0
Speech	3	3	2	0
Rt‐arm outstretched	2	1	0	0
Lt‐arm outstretched	2	1	0	0
Forced vital capacity	3	3	3	2
Rt‐hand grip	3	2	0	0
Lt‐hand grip	3	2	0	0
Head lifted	2	1	0	0
Rt‐leg outstretched	2	1	0	0
Lt‐leg outstretched	2	1	0	0
Total QMG scores	31	23	10	2

Abbreviations: Lt‐: left; QMG, quantitative myasthenia gravis scores; Rt‐, right.

**Figure 3:**
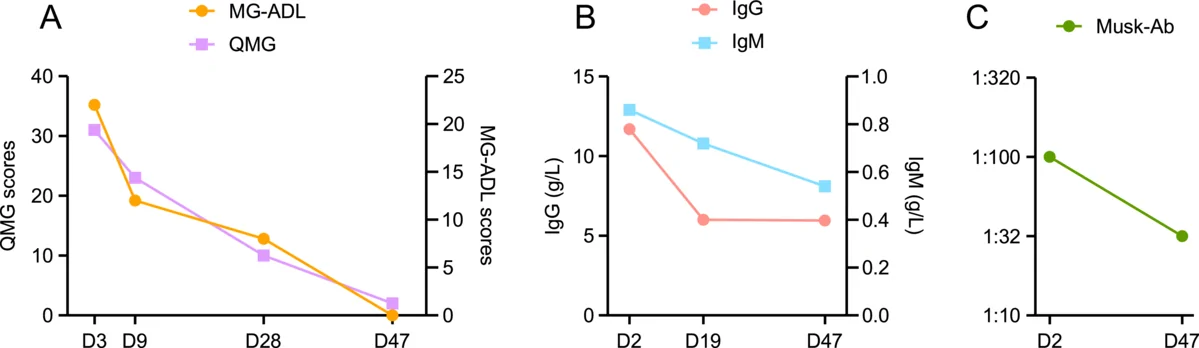
Therapeutic effects of the MuSK‐MG patient. Dynamic changes of QMG and MG‐ADL scores (A), Serum IgG and IgM levels (B), and MuSK‐Ab titers (C) were shown by dates.

## 2 Discussion

MuSK‐MG presents a high risk of disease exacerbations, easily developing into myasthenic crises. In our case, the MuSK‐MG patient was in the state of the impeding myasthenic crisis; rapid and efficient therapy was required to avoid MC. The patient underwent an acute exacerbation of heart failure; IVIG or PLEX was not suitable on account of excessive volume load and a hypercoagulable state. As MuSK antibodies are predominantly of the IgG4 subclass, IVIG is associated with suboptimal efficacy [11]. Moreover, pulmonary infection, osteoporosis, and a history of digestive ulcer made a high dose of glucocorticoids contraindicated for use. Therefore, we considered efgartigimod concurrent with anti–heart failure and antibiotic treatment to be the best plan for the impeding myasthenic crisis.

Mechanistically, efgartigimod resulted in a rapid clearance of IgGs in different subtypes, with IgG4 reduction by 58.1%–66.6% of baseline levels [8]. In a Japanese real‐world study, the responder rate of efgartigimod was higher in the MuSK‐MG patients at 83% compared with 59% in the AChR‐MG patients [12]. Another retrospective study included 14 MuSK‐MG patients treated with efgartigimod, in which MG‐ADL scores of patients decreased significantly from 10.1 ± 4.0 to 2.2 ± 3.1 after the fourth infusion [13]. In phase 3 study of rozanolixizumab, another FcRn inhibitor, 11% of enrolled MG patients were MuSK‐Ab positive, with a responder rate of around 60% [14]. The onset of clinical improvement of efgartigimod is expected to be as early as 1–2 weeks in 70% of enrolled patients, as the ADAPT study reported [9]. In our patient, QMG and MG‐ADL scores showed a remarkable decrease from 31 and 22 to 23 and 12, respectively, after the first infusion of efgartigimod. The fast‐acting effect was consistent with the pivotal clinical trials (ADAPT study); the patient was indicated as a good responder to efgartigimod, and systemic comorbidities of the patient improved gradually under the combination of anti‐infection and anti–heart failure treatment.

For maintenance administration, B cell depletion therapy is recommended as first‐line treatment, and rituximab (RTX) is the most commonly used medication. Whereas the CD20‐specific antibody treatment (RTX) only targets on immature B cells, and does not apply to long‐lived memory B cells or plasmablasts [15, 16]. Some reports have shown that patients with MuSK‐Ab can relapse after several years or not respond to RTX treatment [17, 18, 19]. Furthermore, patients with MG who relapsed after RTX therapy possessed a large percentage (25%) of plasmablasts not expressing CD20. High plasma BAFF (a cytokine promotes B cells differentiation to antibody‐secreting cells, also called BlyS) levels were found in patients with AChR‐MG and MuSK‐MG [19, 20]. The above research results demonstrated the limitation of RTX for MG treatment and provided a promising therapy targeting on inhibition of BAFF or BlyS.

In this instance, telitacicept, the B cell inhibitor by dual BlyS/APRIL blockade, was expected to address the limitation of CD20‐targeted monotherapy. According to its public clinical data, the earliest time of therapeutic effect was 4 weeks after initiation, and 98.1% of patients achieved MSE after 24 weeks of treatment. Our patient achieved MSE within five infusions of telitacicept, occurred earlier than expected, that attributed to the combination use of efgartigimod and telitacicept. The quick clearing of IgG was seen on day 19 in Figure 3, considered to be the action of efgartigimod. Continuous clinical recovery was sustained by repetitive injection of telitacicept, but the effect of IgG levels would lag, just like other B cell targeting therapy. QMG and MG‐ADL scores of the patient showed progressively regression, consistent with the change of MuSK‐ab titers. These meaningful clinical manifestations revealed the potential immunomodulatory effects of telitacicept in B‐cell mediated autoimmune disease. Telitacicept is approved in China for treating systemic lupus erythematosus (SLE) and rheumatoid arthritis (RA). The phase 3 randomized trial of telitacicept in treating MG showed sustained symptom resolution for 24 weeks, accompanied with consistent reduction in serum immunoglobulin levels and CD19‐B cells counts. Despite these promising results, only 4 MuSK‐MG patients were included in the study. More evidence is needed to verify its efficacy in both the acute setting and sustained therapy.

To the best of our knowledge, this is the first case of sequential use of efgartigimod and telitacicept in MuSK‐MG. Their fast‐acting feature, achieving MSE within 1.5 months, and their favorable safety profile, with no observed adverse effects, give us confidence in managing MuSK‐MG. Although the outcome in our patient is encouraging, this report is limited by being an individual medication experience. The optimal dosage and duration regimen is to be explored, and compelling evidence by randomized controlled trials should be validated.

## 3 Conclusion

Our case provided a successful treatment experience by efgartigimod combined with telitacicept in acute phase of very late‐onset MuSK‐MG with severe pulmonary infection and acute heart failure. Further multicenter prospective studies and parallel mechanistic investigations is required to verify the effects of this treatment regimen.

## Author Contributions

**Tianjiao Duan:** writing – original draft, funding acquisition, writing – review and editing, data curation. **Song Ouyang:** funding acquisition, supervision. **Wei Lu:** conceptualization, supervision. **Weifan Yin:** conceptualization, writing – original draft, writing – review and editing.

## Ethics Statement

This study was approved by the Ethics Committee of the Second Xiangya Hospital, Central South University (ethical number: LYF20250200). The patient provided written informed consent for publication.

## Conflicts of Interest

The authors declare no conflicts of interest.

## Data Availability

The data that support the findings of this study are available on request from the corresponding author. The data are not publicly available due to privacy or ethical restrictions.

## References

[1] A. Chaudhuri and P. O. Behan , “Myasthenic Crisis,” QJM: Monthly Journal of the Association of Physicians 102 (2008): 97–107. 10.1093/qjmed/hcn152 19060020

[2] D. B. Sanders , G. I. Wolfe , M. Benatar , et al., “International Consensus Guidance for Management of Myasthenia Gravis: Executive Summary,” Neurology 87 (2016): 419–425. 10.1212/WNL.0000000000002790 PMC4977114 27358333

[3] A. G. Vakrakou , E. Karachaliou , E. Chroni , et al., “Immunotherapies in MuSK‐Positive Myasthenia Gravis; An IgG4 Antibody‐Mediated Disease,” Frontiers in Immunology 14 (2023): 1212757. 10.3389/fimmu.2023.1212757 PMC10410455 37564637

[4] S. S. Luo , H. Zhou , Y. X. Yue , et al., “Expert Consensus on the Management of Impending Myasthenic Crisis,” Chinese Journal of Clinical Neurosciences 32, no. 3 (2024): 241–251.

[5] C. Rodolico , C. Bonanno , A. Toscano , and G. Vita , “MuSK‐Associated Myasthenia Gravis: Clinical Features and Management,” Frontiers in Neurology 11 (2020): 660. 10.3389/fneur.2020.00660 PMC7390870 32793097

[6] R. Iorio , V. Damato , P. E. Alboini , and A. Evoli , “Efficacy and Safety of Rituximab for Myasthenia Gravis: A Systematic Review and Meta‐Analysis,” Journal of Neurology 262 (2015): 1115–1119. 10.1007/s00415-014-7532-3 25308632

[7] Z. Bi , Y. Li , J. Lin , M. Gui , Z. Li , and B. Bu , “Long‐Term Efficacy and Safety of Tacrolimus in Anti‐MuSK Antibody‐Positive Myasthenia Gravis: A Retrospective Single‐Center Cohort Study,” Neurological ScienceS 46 (2025): 943–949. 10.1007/s10072-024-07819-8 39503950

[8] P. Ulrichts , A. Guglietta , T. Dreier , et al., “Neonatal Fc Receptor Antagonist Efgartigimod Safely and Sustainably Reduces IgGs in Humans,” Journal of Clinical Investigation 128 (2018): 4372–4386. 10.1172/JCI97911 PMC6159959 30040076

[9] J. F. Howard, Jr. , V. Bril , T. Vu , et al., “Safety, Efficacy, and Tolerability of Efgartigimod in Patients With Generalised Myasthenia Gravis (ADAPT): A Multicentre, Randomised, Placebo‐Controlled, Phase 3 Trial,” Lancet Neurology 20 (2021): 526–536. 10.1016/S1474-4422(21)00159-9 34146511

[10] F. Shi , R. Xue , X. Zhou , P. Shen , S. Wang , and Y. Yang , “Telitacicept as a BLyS/April Dual Inhibitor for Autoimmune Disease,” Immunopharmacology and Immunotoxicology 43 (2021): 666–673. 10.1080/08923973.2021.1973493 34519594

[11] R. L. Hurst and C. L. Gooch , “Muscle‐Specific Receptor Tyrosine Kinase (MuSK) Myasthenia Gravis,” Current Neurology and Neuroscience Reports 16 (2016): 61. 10.1007/s11910-016-0668-z 27170368

[12] S. Suzuki , A. Uzawa , Y. Nagane , et al., “Therapeutic Responses to Efgartigimod for Generalized Myasthenia Gravis in Japan,” Neurology: Clinical Practice 14 (2024): e200276. 10.1212/CPJ.0000000000200276 PMC10965358 38544885

[13] Y. Zhou , Q. Zhou , Y. Yue , et al., “Efgartigimod for Induction and Maintenance Therapy in Muscle‐Specific Kinase Myasthenia Gravis,” Therapeutic Advances in Neurological Disorders 18 (2025): 17562864251326778. 10.1177/17562864251326778 PMC11938440 40144041

[14] A. A. Habib , S. Sacconi , G. Antonini , et al., “Efficacy and Safety of Rozanolixizumab in Patients With Muscle‐Specific Tyrosine Kinase Autoantibody‐Positive Generalised Myasthenia Gravis: A Subgroup Analysis of the Randomised, Double‐Blind, Placebo‐Controlled, Adaptive Phase III Mycaring Study,” Therapeutic Advances in Neurological Disorders 17 (2024): 17562864241273036. 10.1177/17562864241273036 PMC11409299 39297052

[15] P. Stathopoulos , A. Kumar , J. A. V. Heiden , E. Pascual‐Goñi , R. J. Nowak , and K. C. O'Connor , “Mechanisms Underlying B Cell Immune Dysregulation and Autoantibody Production in MuSK Myasthenia Gravis,” Annals of the New York Academy of Sciences 1412 (2018): 154–165. 10.1111/nyas.13535 PMC5793885 29381221

[16] P. Stathopoulos , A. Kumar , R. J. Nowak , and K. C. O'Connor , “Autoantibody‐Producing Plasmablasts After B Cell Depletion Identified in Muscle‐Specific Kinase Myasthenia Gravis,” JCI Insight 2. no. 17 (2017): e94263. 10.1172/jci.insight.94263 PMC5621905 28878127

[17] E. Cortés‐Vicente , R. Rojas‐Garcia , J. Díaz‐Manera , et al., “The Impact of Rituximab Infusion Protocol on the Long‐Term Outcome in Anti‐MuSK Myasthenia Gravis,” Annals of Clinical and Translational Neurology 5 (2018): 710–716. 10.1002/acn3.564 PMC5989782 29928654

[18] M. L. Fichtner , K. B. Hoehn , E. E. Ford , et al., “Reemergence of Pathogenic, Autoantibody‐Producing B Cell Clones in Myasthenia Gravis Following B Cell Depletion Therapy,” Acta Neuropathologica Communications 10 (2022): 154. 10.1186/s40478-022-01454-0 PMC9617453 36307868

[19] S. Y. Kang , C. H. Kang , and K. H. Lee , “B‐Cell‐Activating Factor Is Elevated in Serum of Patients With Myasthenia Gravis,” Muscle & Nerve 54 (2016): 1030–1033. 10.1002/mus.25162 27121160

[20] F. Scuderi , P. E. Alboini , E. Bartoccioni , and A. Evoli , “BAFF Serum Levels in Myasthenia Gravis: Effects of Therapy,” Journal of Neurology 258 (2011): 2284–2285. 10.1007/s00415-011-6092-z 21559937

